# Cyclic Voltammetry Study of Noble Metals and Their Alloys for Use in Implantable Electrodes

**DOI:** 10.1021/acsomega.2c03563

**Published:** 2022-09-13

**Authors:** Megan K. Puglia, Patrick K. Bowen

**Affiliations:** Research & Development, Deringer-Ney, Inc., 353 Woodland Ave., Bloomfield, Connecticut 06002, United States

## Abstract

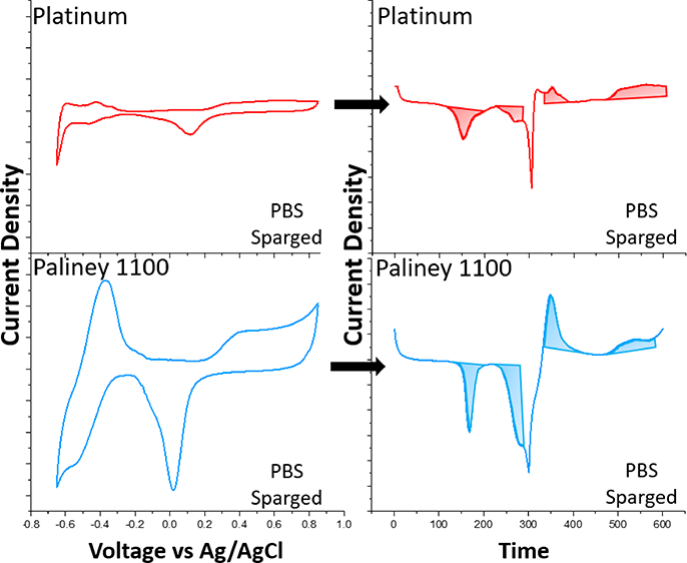

Innovation in the application and miniaturization of implantable electrodes has caused a spike in new electrode material research; however, few robust studies are available that compare different metal electrodes in biologically relevant media. Herein, cyclic voltammetry has been employed to compare platinum, palladium, and gold-based electrodes’ potentiometric scans and their corresponding charge storage capacities (CSCs). Ten different noble metals and alloys in these families were tested under pseudophysiological conditions in phosphate-buffered saline (pH 7.4) at 37 °C. Charge storage capacity values (mC/cm^2^) were calculated for the oxide reduction, hydrogen adsorption, hydrogen desorption, and oxide formation peaks. Five scan rates spanning 2 orders of magnitude (10, 50, 100, 500, and 1000 mV/s) in both sparged and aerated environments were evaluated. Materials have been ranked by their charge storage capacities, reversibility, and trends discussed. Palladium-based alloys outperformed platinum-based alloys in the sparged condition and were ranked equally as high in the aerated condition. The Paliney 1100 (Pd-Re) alloy gave the highest observed calculated CSC value of 0.64 ± 0.02 mC/cm^2^ in the aerated condition, demonstrating 73 ± 5% reversibility. Trends between metal electrode families elicited in this study can afford valuable insight into future engineering of high performing implantable electrode materials.

## Introduction

Since the introduction of the cochlear implant in the 1960s, tissue-contacting electrodes (TCEs) have been successfully employed in a variety of medical treatments. Today, there are a number of clinically approved implantable electrode systems available on the market for the treatment of a myriad of medical conditions using both tissue stimulation and signal recording devices. Applications include the treatment of epilepsy, Parkinson’s disease, dystonia, and depression through deep brain stimulation (DBS) as well as depression and epilepsy through vagus nerve stimulation.^1^ Nervous system electrode-prostheses systems assist with auditory and visual impairments and spinal cord injuries and can record signaling for the use of assistive devices and muscle stimulators.^1^ Other applications for TCEs include addiction management,^2^ paralysis,^3^ and chronic pain management.^4^ The sustained clinical success of these devices has spawned a new era of material science research in pursuit of superior implantable electrode materials.

There are a number of characteristics to consider in designing a bioelectrode. Implantable electrodes must maintain biocompatibility with their intended system as well as long-term stability within that system. The material must be manufacturable to clinically relevant shapes and sizes while also maintaining appropriate mechanical properties dictated by the assembly processes and applications. High material charge injection limits and low impedance values are desirable; these traits allow for the fabrication of smaller electrodes and reduced noise in signaling devices.^5^

The consideration of chemical interactions occurring at the material-tissue interface is imperative for electrode success. Understanding these interactions affords insight into system pH changes, the potential for material dissolution and resultant ion release, safe potential windows for use, charge densities, and overall electrode performance.^6^ Knowledge of the maximum reversible charge injection capacity (CIC) an electrode can inject as well as the types of electrochemical reactions occurring at the electrode surface is imperative in designing safe electrode materials.

Cyclic voltammetry (CV) experiments allow researchers to study chemical reactions occurring at the electrode surface in different environments by subjecting the working electrode to scanning potentials and recording the resultant current. CV experiments give the appropriate potential windows for specific systems that do not cause gas evolution or chemical byproducts as well as indicate the reversibility of the electrochemical reactions occurring at the electrode-system interface. CV scans also allow the calculation of an electrode’s charge storage capacity (CSC). CSC is a charge density value that measures an electrode’s ability to store charge per its surface area, typically expressed in mC/cm^2^. CSC is the integral of current produced over time as the voltage is varied over the water window in a typical CV experiment. The portion of the CSC that is available for reversible charge injection during pulsing is the aforementioned CIC.^7^ Therefore, a larger CSC can often lead to a larger CIC in the same potential window, a benefit for tissue stimulation applications.^5,8−10^ Higher CIC values allow for smaller electrode surfaces that can safely achieve higher current densities at lower potentials in devices.^11^ CV experiments often over-estimate CSC due to their slower potential scan rates than in real application. The lower rate of potential change allows electrochemical reactions to complete within smaller potential ranges. However, the relationship between CSC and CIC for the same electrode allows for the use of CSC calculations as a simple and efficient way to rank materials according to their potential CIC value.^12^

**Figure 1:**
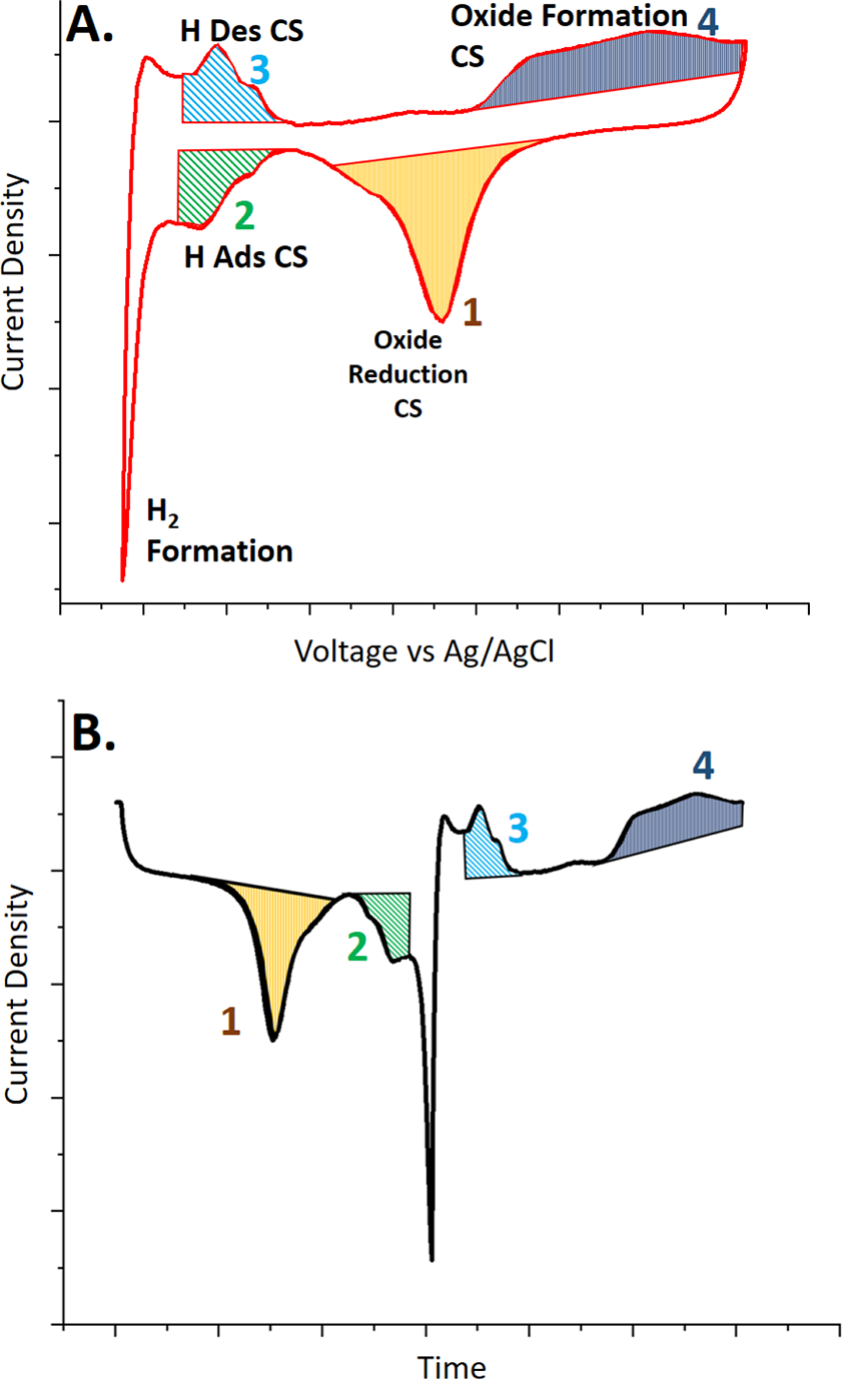
(A) Peak areas used for the CSC calculations of different electrochemical peaks. CSCs were calculated using the integrated area of the current vs time plot peaks (B). Peak numbers in panel (B) correspond to peak areas in the CV plot.

**Figure 2:**
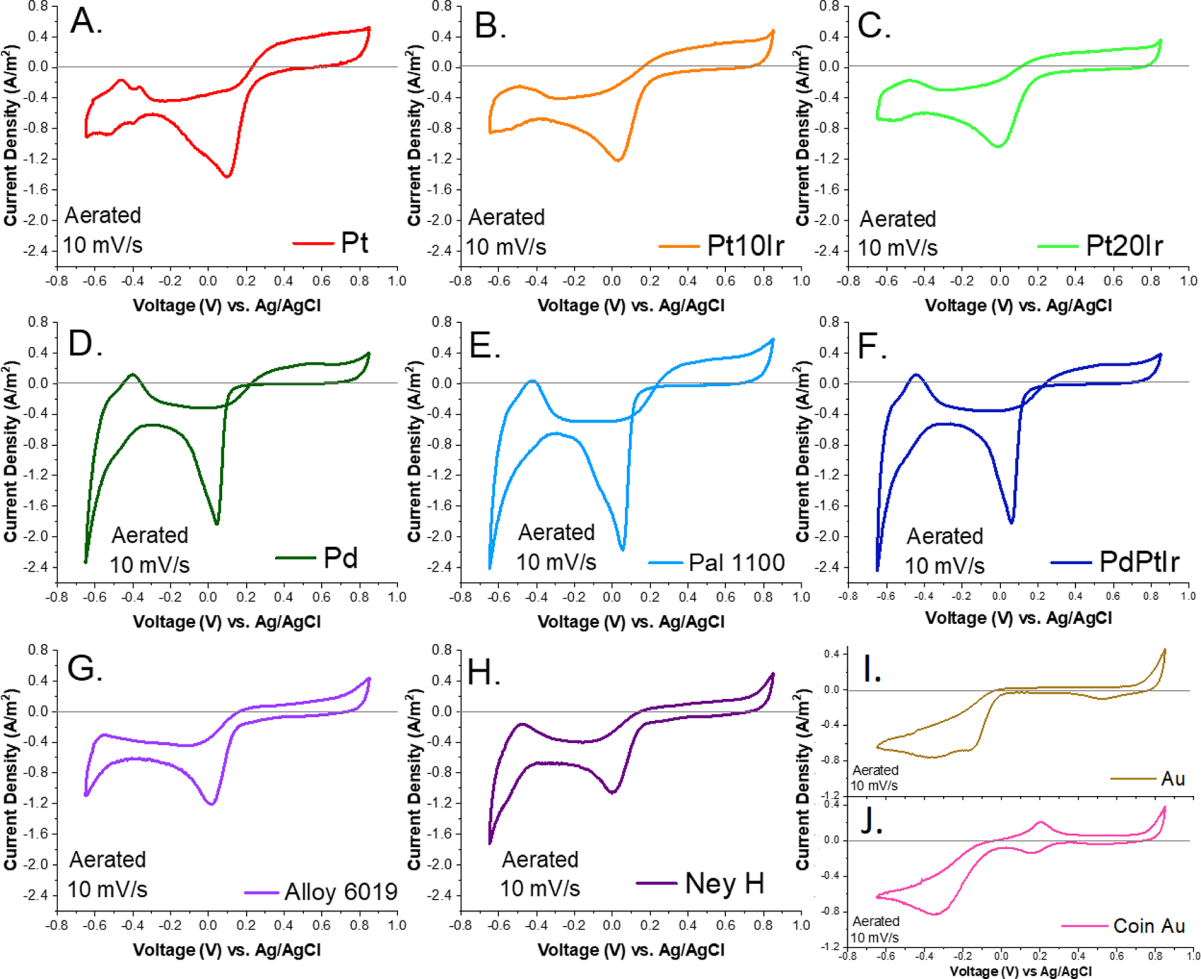
CV scans in PBS (pH = 7.4) under aerated conditions using a 10 mV/s scan rate for electrodes made with Pt (A), Pt10Ir (B), Pt20Ir (C), Pd (D), Pal 1100 (E), PdPtIr (F), Alloy 6019 (G), Ney H (H), Au (I), and Coin Au (J). Note the *y*-axis scale differences for the Au and Coin Au plots.

**Figure 3:**
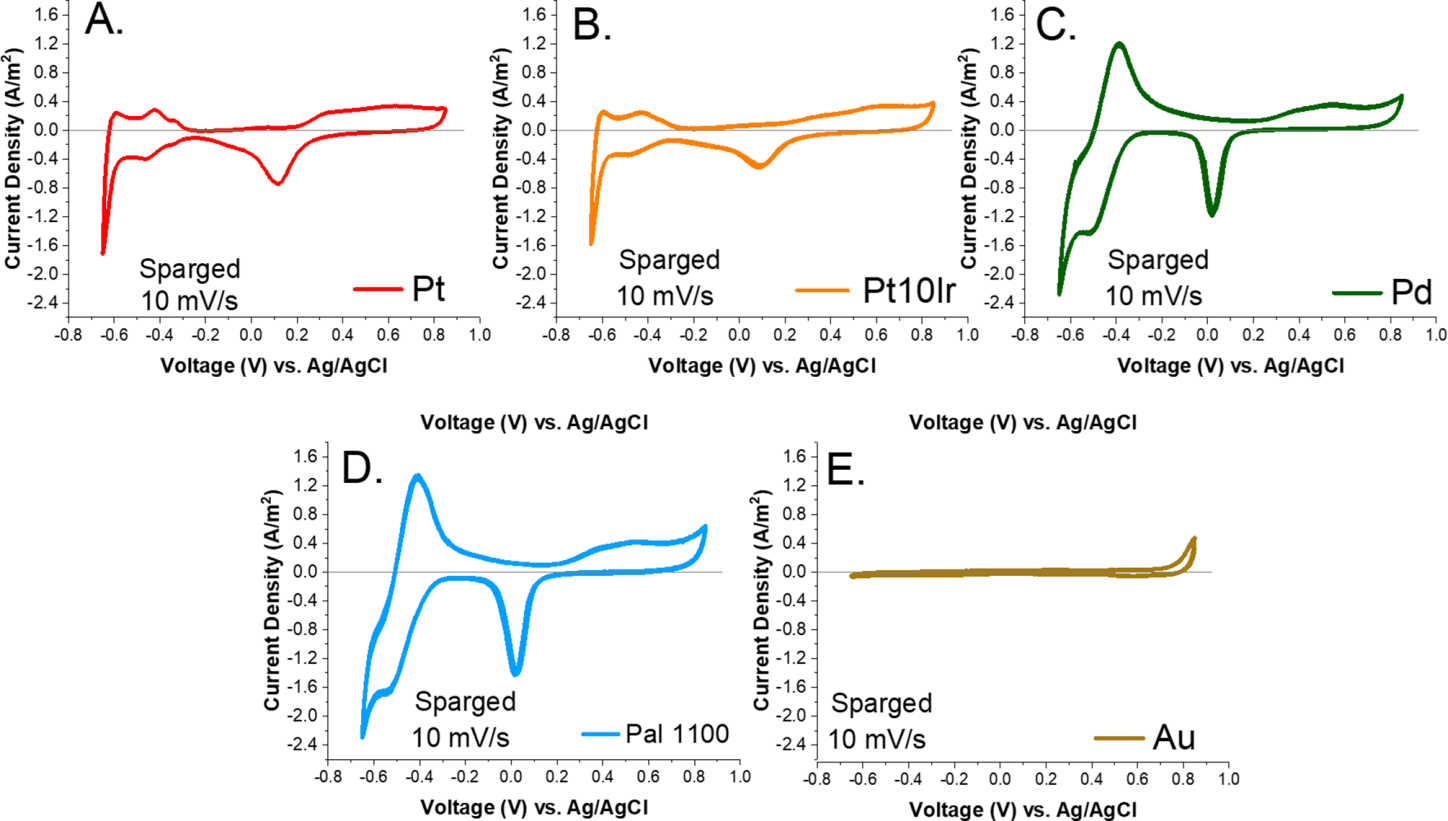
CV scans in PBS (pH = 7.4) under sparged conditions using a 10 mV/s scan rate for electrodes made with Pt (A), Pt10Ir (B), Pd (C), Pal 1100 (D), and Au (E).

**Figure 4:**
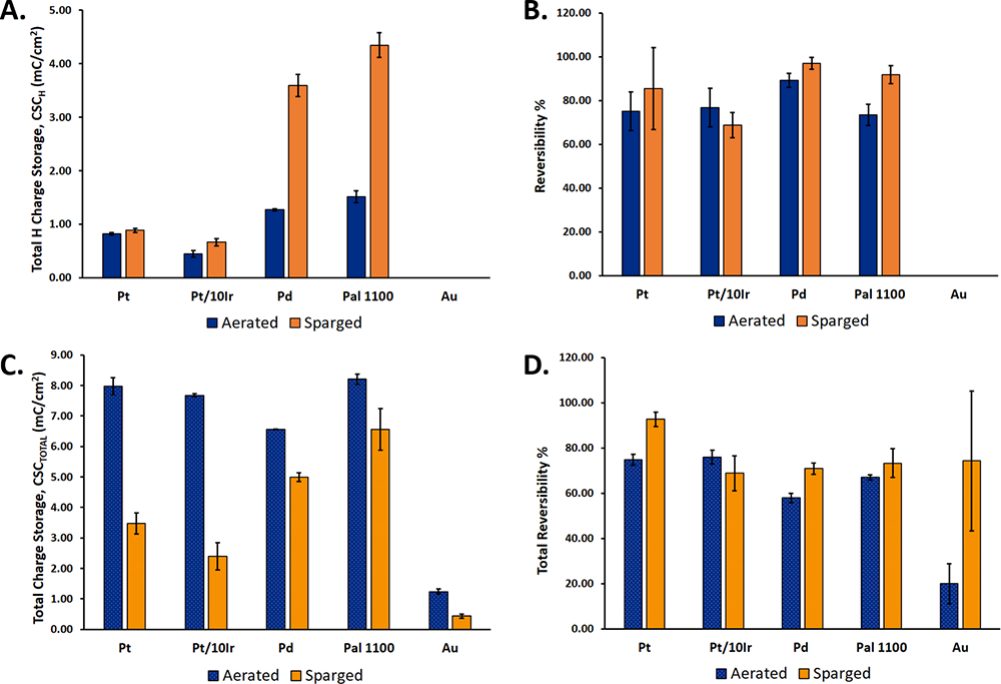
Comparison of CSC_H_ (A), H reversibility % (B), CSC_TOTAL_ (C), and total reversibility % (D) under sparged (orange) and aerated (navy) conditions for Pt, Pt10Ir, Pd, Pal 1100, and Au electrodes.

A schematic CV scan of a platinum (Pt) electrode in sparged phosphate-buffered saline, pH = 7.4, (PBS) is shown in Figure 1 where the well-established oxide reduction (Figure 1, 1), hydrogen adsorption (Figure 1, 2), hydrogen desorption (Figure 1, 3), and oxide formation (Figure 1, 4) regions have been labeled. The sequence of labeling begins from the maximum potential in the cathodic sweep.

The first reaction observed during the sweep to increasingly cathodic potentials is oxide reduction (Figure 1, 1). In this regime, the metal surface becomes progressively less electron-deficient, and platinum oxides and hydroxides return borrowed electrons to the medium. Some of the known reversible electrochemical reactions on the platinum surface are shown below in eqs 1 and 2.^13^ The regime of oxide reduction will be abbreviated as Oxide_Red_ in the present report.
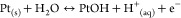
1
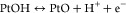
2

The second and third reactions observed in the Pt cyclic voltammogram relate to hydrogen adsorption (Figure 1, 2) and hydrogen desorption (Figure 1, 3) phenomena, respectively. The reversible equation representing this reaction on Pt is shown below as eq 3. As the voltage is swept in the cathodic direction (potential is decreased), an electron is transferred from the Pt electrode to a proton (H^+^), which becomes adsorbed to a single site on the Pt surface. The adsorption region is abbreviated H_ads_. Once the Pt surface is completely covered with adsorbed H, further cathodic electron transfer will begin to create hydrogen gas (H_2_).^12^ When the potential is increased in the anodic direction, the reverse reaction occurs and the H^+^ desorbs from the Pt surface, and this regime is designated H_des_.
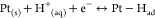
3

This is the primary mechanism of charge transfer and storage on many noble metal bioelectrodes.^12^ The reaction is a kinetically fast redox reaction and is characterized as pseudo-capacitance. Reversible electrochemical reactions such as this are preferred for tissue stimulation because they produce no electrochemical byproducts.

After H_des_, the potential is continually increased in the anodic direction and an oxide formation peak (Figure 1, 4) is observed as hydroxyl species in the medium donate an electron to the electron-deficient Pt surface. This is described in the aforementioned eqs 1 and 2 and abbreviated as Oxide_Form_. These processes are often diffusion-controlled at a metal electrode surface; however, each unique system must be studied to determine the governing reaction kinetics. When a reaction is diffusion-limited, its peak current is proportional to the square root of the scan rate as determined by the Randles–Ševčik equation.^14^

The CSC values corresponding to the individual reactions—signified by shaded peak areas in Figure 1A**—**are calculated by taking the integral of the peak areas shown in the corresponding current density vs time plot (Figure 1B).

Noble metals, such as platinum (Pt) and gold (Au), have a successful history in implantable electrode systems due to both possessing electrical conductivity, resistance to corrosion, stability, and well-studied mechanical behavior. Functionalization and coating of noble metal surfaces are not required for compatibility in specific biological systems and are not commonly used in clinically available implantable electrode systems.

Many implantable electrode systems available on the market are made with Pt or higher hardness platinum/iridium alloys (Pt-Ir). These include the Vercise DBS system (Pt-Ir, Boston Scientific), SENSIGHT Directional Leads for DBS and sensing (Pt-Ir, Medtronic), LivaNova Vegas nerve stimulation therapy system (Pt-Ir, LivaNova), and FLEX Series Electrode arrays for cochlear implants (Pt, Pt-Ir, and MED-EL).^15−18^

Reported literature values for of the CSC of Pt range widely, from 0.55 to 6.1 mC/cm^2^.^8,12,19^ Reported CSC values for Pt-Ir range from 4.6 to 128.2 mC/cm^2^.^20^ CSC values for Au and palladium (Pd) are increasingly difficult to find in the literature with one report giving a CSC value for gold of 0.32 mC/cm^2^.^21^ The wide range of values that are reported reflect variations in electrode size, scan rate, scanned region, and electrode testing environment. The lack of available information as well as lack of experimental consistency and information on alloys makes fair comparison between metal electrode materials difficult. To develop meaningful comparisons between electrode materials, the CV curves of 10 different metals and alloys were studied at multiple scan rates, under the same experimental conditions, in both aerated and sparged environments.

## Methods and Materials

### Materials

Raw metallic elements and purities utilized in the present study included gold (99.99%+), copper (99.99%+), iridium (99.95%+), palladium (99.95%+), platinum (99.95%+), and rhenium (99.99%+). Per U.S. HR 4173, Section 1502, Dodd-Frank Wall Street Reform and Consumer Protection Act, due diligence has been undertaken to confirm that the supply of the aforementioned metals does not originate from the Democratic Republic of Congo or adjoining countries.

Isopropyl alcohol was sourced from Thermo Fisher Scientific (Ward Hill, Massachusetts). Phosphate-buffered saline (10× solution, Fisher Bioreagents) was purchased from Thermo Fisher Scientific (Fair Lawn, New Jersey). Silicon carbide grinding papers were procured from Lapmaster Wolters (Mount Prospect, Illinois).

### Electrode Preparation

Nominal compositions of the alloys used in the present study are presented in Table 1. Certain alloys conformed to their respective ASTM International standard compositional requirements including Au 99.5 (B562), Coin Gold (B596), and Pt-10 wt % Ir (B684). Neyoro H is detailed in U.S. Patent 8,845,959,^22^ Paliney 1100 in 7,354,488.^23^ Specific electrode chemistry analyses are compiled in Table S1 in the Supporting Information.

**Table 1:** Nominal Alloy Constituents of the Gold-, Platinum-, and Palladium-Based Alloys

		nominal composition (wt %)					
	abbreviation used	Au	Cu	Ir	Pd	Pt	Re
gold-based alloys							
gold	Au	100					
coin gold	Coin Au	90	10				
Alloy 6019	6019	60		1	20	19	
Neyoro H	Ney H	58		1	31	10	
platinum-based alloys							
platinum	Pt					100	
platinum-10 wt % iridium	Pt10Ir			10		90	
platinum-20 wt % iridium	Pt20Ir			20		80	
palladium-based alloys							
palladium	Pd				100		
Paliney 1100	Pal 1100				90.5		10.5
palladium-20 wt % platinum-1 wt % iridium	PdPtIr			1	79	20	

Raw metals were weighed in the proper proportions. Metals were then charged into either clay silica, fused silica, graphite, or yttria-stabilized zirconia crucible according to the composition. The charge was then heated by induction under a carbon monoxide flame and cast into a graphite mold. Cast billets were reduced in size by rod milling on hardened steel rolls and wire drawing through carbide dies using proprietary heat treatment and cold work schedules.

When rods reached a size of ∼5 mm diameter, electrodes were lathe-turned to a nominal dimension of 4.75 mm diameter × 25 mm long, and threads were cut into one end. The turned surfaces were affixed to a rotary tool and dry-sanded with progressively finer silicon carbide abrasive papers—320-, 400-, and 600-grit—to a final 1200-grit SiC finish. Finally, the electrodes were cleaned with deionized water and isopropyl alcohol.

### Surface Roughness Measurement

The surface quality of the electrodes was evaluated on a Wyko Veeco NT white light interferometer equipped with Wyko Vision32 v. 2.210 PC software. The vertical scan interferometry (VSI) mode was used at 10.4× nominal magnification, 0.58 × 0.46 mm field of view, and 480 × 736 pixel image size. Cylinder and tilt corrections were applied. The arithmetic mean roughness (*R*_a_) was calculated across the acquired fields of view. For a typical interferometer output, refer to Figure S1.

### Cyclic Voltammetry Experiments

Care was taken in cyclic voltammetry experimental design to closely follow testing guidelines put forth in ASTM F2129-04 and ASTM G5-94.

A wavenowXV potentiostat station and corresponding Aftermath software (AfterMath Data Organizer V1.2.3239, Pine Research Instrumentation) were used to conduct all electrochemistry experiments. A three-electrode cell was set up in a 1 L glass chamber equipped with water jacket and multiple ports for the working electrode, counter electrode, reference electrode, thermometer, and gas line inlet and outlets. The cell was filled with 1× phosphate-buffered saline (PBS) (pH = 7.4) for all electrochemistry measurements. The PBS comprised 137 mM NaCl, 2.7 mM KCl, 10 mM Na_2_HPO_4_, and 1.8 mM KH_2_PO_4_.

Current flowed between the sample working electrode and a 0.64 cm diameter × 10.48 cm long graphite counter electrode. Electrode potentials were measured with respect to a Ag/AgCl reference electrode in 3 M KCl using a Luggin capillary within close proximity to, and pointed toward, the working electrode. Threaded working electrodes were screwed onto the end of a tapped stainless steel rod housed inside a plastic holder with the junction sealed using an O-ring, thus forming the working electrode.

For all experiments, the glass chamber was filled with 0.65 L of PBS and allowed to equilibrate at 37 °C for 30 min prior to start. For sparged experiments, argon gas was bubbled vigorously through the solution for 30 min prior to the experiment to minimize dissolved oxygen. Argon gas bubbling was continued through experimentation at a greatly reduced flow rate. No argon gas was bubbled before, or during cycling, for the aerated experiments.

Prior to experimentation, working electrodes were rinsed with isopropyl alcohol and dried. All working electrodes were cycled from +0.85 to −0.65 V, the average reported water window for PBS (pH = 7.4).^24^ Each electrode was cycled 50 times at 1 V/s to reach a steady state, and then, five complete cycles (cathodic + anodic sweep) were done at 1000, 500, 100, 50, and 10 mV/s scan rates consecutively. Calculations were done using the final electrode cycle for each scan rate. At least three trials were run for each electrode material at different times, using multiple fabricated electrodes when available. CV scans shown are the average of at least three trials between electrodes of each material.

### Charge Storage Calculations

CSC values were calculated by taking the integral of each peak in the current versus time graph that resulted from each CV scan at each scan rate using Aftermath software (AfterMath Data Organizer V1.2.3239, Pine Research Instrumentation). Baselines were drawn at the scan state prior to each peak using linear regression models in the aftermath software, apart from the hydrogen desorption peak where the baseline was drawn at the steady state immediately following the peak. Care was taken such that hydrogen evolution reactions and oxygen evolution reactions were excluded from integration calculations where needed. Additional values were calculated from the sum of specific peak’s CSC values as indicated below:
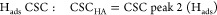
4
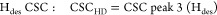
5
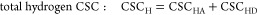
6
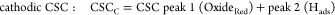
7
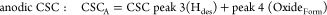
8
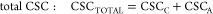
9
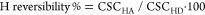
10
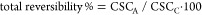
11

### Statistical Methods

Statistical method experiments were conducted in triplicate. Unless stated otherwise, reported errors and error bars are the sample standard deviation of the three measurements.

## Results

CV scans for each material completed under aerated conditions at 10 mv/s are shown in Figure 2. With the exception of Au and Coin Au, each material scan exhibited the aforementioned characteristic regions: oxide reduction, hydrogen adsorption, hydrogen desorption, and oxide formation. Pd-containing alloys also showed hydrogen formation and oxygen formation peaks at the outer bounds of the potential scan range.

The CSC value for the peaks of each scan for each material was calculated via integration of current vs time in a manner similar to that illustrated in Figure 1B. Baselines were drawn for each peak calculation as indicated in the Materials and Methods section. Care was taken so as not to overestimate charge storage capacities for any one chemical reaction and to account for changes in baseline due to environmental factors, such as sparging versus aeration. As such, potential limits for peak integration were different for each material depending on the reaction onset potential for each material.

CSC measurements for each material in the aerated condition are shown in Table 2. For the Au and Coin Au electrodes, specific H_ads_ and H_des_ peaks could not be distinguished, so only cathodic and anodic CSC values were calculated using 0.0 A/m^2^ as the baseline. These values were added to calculate a CSC_TOTAL_ and divided to calculate the total reversibility, but other parameters could not be determined.

**Table 2:** Summary of All Calculations Done for Each Electrode Material under the Aerated Condition at 10 mV/s^a^

electrode material	surface roughness *R*_a_ (nm)	CSC_HA_ (mC/cm^2^)	CSC_HD_ (mC/cm^2^)	CSC_H_ (mC/cm^2^)	H reversibility (%)	CSC_C_ (mC/cm^2^)	CSC_A_ (mC/cm^2^)	CSC_TOTAL_ (mC/cm^2^)	total reversibility (%)
Pt	146 ± 15	–0.34 ± 0.03	0.45 ± 0.04	0.79 ± 0.05	75.0 ± 8.9	–4.56 ± 0.22	3.41 ± 0.06	7.98 ± 0.28	74.9 ± 2.4
Pt10Ir	137 ± 2	–0.23 ± 0.01	0.21 ± 0.06	0.45 ± 0.06	76.7 ± 8.8	–4.36 ± 0.09	3.31 ± 0.07	7.68 ± 0.05	76.0 ± 3.1
Pt20Ir	106	–0.16 ± 0.01	0.21 ± <0.01	0.37 ± 0.02	79.9 ± 6.1	–4.18 ± 0.30	2.70 ± 0.39	6.88 ± 0.69	64.2 ± 4.9
Pd	124 ± 21	–0.60 ± <0.01	0.67 ± 0.02	1.27 ± 0.02	89.1 ± 3.1	–4.15 ± 0.05	2.41 ± 0.06	6.56 ± 0.01	58.0 ± 2.0
Pal 1100	117 ± 7	–0.64 ± 0.02	0.88 ± 0.09	1.52 ± 0.11	73.3 ± 4.8	–4.92 ± 0.13	3.29 ± 0.04	8.21 ± 0.17	67.0 ± 1.1
PdPtIr	127 ± 31	–0.55 ± 0.02	0.71 ± 0.04	1.26 ± 0.03	77.8 ± 6.1	–3.98 ± 0.10	2.88 ± 0.08	6.87 ± 0.18	72.3 ± 0.6
Alloy 6019	107 ± 11	–0.15 ± 0.01	0.16 ± 0.02	0.31 ± 0.02	83.2 ± 3.3	–3.72 ± 0.14	1.53 ± 0.08	5.26 ± 0.19	41.1 ± 2.3
Ney H	113 ± 8	–0.42 ± 0.02	0.32 ± 0.09	0.74 ± 0.09	73.5 ± 17.9	–2.67 ± 0.50	1.55 ± 0.06	4.22 ± 0.47	60.4 ± 13.6
Au	179 ± 42	N/A	N/A	N/A	N/A	–1.03 ± 0.10	0.20 ± 0.08	1.23 ± 0.08	20.0 ± 8.8
Coin Au	117 ± 13	N/A	N/A	N/A	N/A	–3.18 ± 0.05	0.41 ± 0.04	3.59 ± 0.01	13.1 ± 1.6

a Only one Pt20Ir electrode was made, so there is not a surface roughness standard deviation to report.

The Pal 1100 electrode gave the highest CSC_H_ (1.52 ± 0.11 mC/cm^2^) followed by Pd (1.27 ± 0.02 mC/cm^2^), PdPtIr (1.26 ± 0.03 mC/cm^2^), and Pt (0.79 ± 0.05 mC/cm^2^).

These results may be considered baseline values for these metals and alloys as they utilize typically low CV scan rates in an oxygen-containing environment. Results from other experimental permutations using a hypoxic environment and increasing the CV scan rate are detailed in the following sections.

### Results from a Sparged Environment

To better understand how the CV scans of each material were affected by the presence of oxygen, a subset of the materials was run after sparging the cell with argon (the “sparged” condition) to remove dissolved O_2_. These data are shown in Figure 3. Under the sparged condition, Pt, Pt10Ir, Pd, and Pal 1100 all show an increase in the H_2_ formation peak. The same calculations were completed for each material run in the sparged condition, and the results are shown in Table 3. Interestingly, again, Pal 1100 and Pd showed the highest total CSC_H_ (4.35 ± 0.23 and 3.59 ± 0.21 mC/cm^2^, respectively), followed by Pt (0.88 ± 0.03 mC/cm^2^), and then Pt10Ir (0.67 ± 0.07 mC/cm^2^). Under the sparged condition, the baseline for all CV scans remained steadily close to zero current density, in contrast to the scans under aerated conditions, which showed negative baseline values for the oxygen reduction peak and the H adsorption peaks.

**Table 3:** Summary of All Calculations Done for Each Electrode under the Sparged Condition at 10 mV/s

electrode material	surface roughness *R*_a_ (nm)	CSC_HA_ (mC/cm^2^)	CSC_HD_ (mC/cm^2^)	CSC_H_ (mC/cm^2^)	H reversibility (%)	CSC_C_ (mC/cm^2^)	CSC_A_ (mC/cm^2^)	CSC_TOTAL_ (mC/cm^2^)	total reversibility (%)
Pt	146 ± 15	–0.40 ± 0.05	0.48 ± 0.08	0.88 ± 0.03	85.4 ± 18.6	–1.74 ± 0.14	1.73 ± 0.23	3.47 ± 0.35	92.7 ± 3.2
Pt10Ir	137 ± 2	–0.27 ± 0.04	0.39 ± 0.03	0.67 ± 0.07	68.8 ± 5.7	–1.43 ± 0.33	0.96 ± 0.13	2.40 ± 0.45	68.9 ± 7.8
Pd	124 ± 21	–1.81 ± 0.13	1.78 ± 0.09	3.59 ± 0.21	96.9 ± 2.7	–2.92 ± 0.09	2.07 ± 0.07	4.99 ± 0.14	70.9 ± 2.6
Pal 1100	117 ± 7	–2.27 ± 0.11	2.08 ± 0.14	4.35 ± 0.23	91.7 ± 4.2	–3.80 ± 0.52	2.76 ± 0.20	6.56 ± 0.69	73.3 ± 6.4
Au	179 ± 42	N/A	N/A	N/A	N/A	–0.18 ± 0.07	0.25 ± 0.03	0.43 ± 0.07	74.3 ± 30.9

Figure 4 compares the results of electrode materials done under both aerated and sparged conditions. Figure 4A plots the CSC_H_ for Pt, Pt10Ir, Pd, Pal 1100, and Au in both aerated and sparged conditions. The CSC_H_ value for each material is higher under the sparged condition; however, rankings of CSC_H_ between alloys (Pal 1100 > Pd > Pt > Pt10Ir) remain the same for both conditions, with Pal 1100 giving the highest values (1.52 ± 0.11 mC/cm^2^ aerated and 4.35 ± 0.23 mC/cm^2^ sparged).

Figure 4B shows the H reversibility % for electrodes done under both sparged and aerated conditions. The percent differences between H reversibility % values calculated from the aerated and sparged conditions of each material were all lower than 20%. The average H reversibility % under aerated conditions was 78.5 ± 6.2%, and the sparged average was 85.7 ± 10.6%. Overall, Pd shows the highest H reversibility % under the aerated condition (89.1 ± 3.1%) with all other materials performing within error of each other. Under sparged conditions, Pd (96.9 ± 2.7%) again shows the highest H reversibility % with Pal 1100 (91.7 ± 4.2%), Pt (85.4 ± 18.6%), and Pt10Ir (68.8 ± 5.7%) ranking below.

Figure 4C shows the CSC_TOTAL_ for each material done under aerated vs sparged conditions. Conversely from CSC_H_, the aerated condition shows higher values for CSC_TOTAL_ than the sparged condition. Aerated Pal 1100 (8.21 ± 0.17 mC/cm^2^) ranks the highest followed closely by Pt (7.98 ± 0.28 mC/cm^2^), Pt10Ir (7.68 ± 0.05 mC/cm^2^), and Pd (6.56 ± 0.01 mC/cm^2^). Au (1.23 ± 0.08 mC/cm^2^) had the lowest aerated CSC_TOTAL_ value. The sparged condition does not show the same trend for the CSC_TOTAL_ value with Pal 1100 (6.56 ± 0.69 mC/cm^2^), again giving the highest value but Pd (4.99 ± 0.14 mC/cm^2^) ranking second, with Pt (3.47 ± 0.35 mC/cm^2^), Pt10Ir (2.40 ± 0.45 mC/cm^2^), and Au (0.43 ± 0.07 mC/cm^2^) thereafter.

Figure 4D shows the reversibility of CSC_TOTAL_ for all electrode materials under both conditions. Under aerated conditions, Pt10Ir (76.0 ± 3.1%) gives the highest total reversibility % values, closely followed by Pt (74.9 ± 2.4%), and then Pal 1100 (67.0 ± 1.1%) and Au (20.0 ± 8.8%). All but Pt10Ir show a higher total reversibility % under the sparged condition. Pt (92.7 ± 3.2%) shows the highest sparged total reversibility % value followed by Au (74.3 ± 30.9%), Pal 1100 (73.3 ± 6.4%), Pd (70.9 ± 2.6%), and Pt10Ir (68.9 ± 7.8%).

### Results from Varying Voltage Sweep Rates

**Figure 5:**
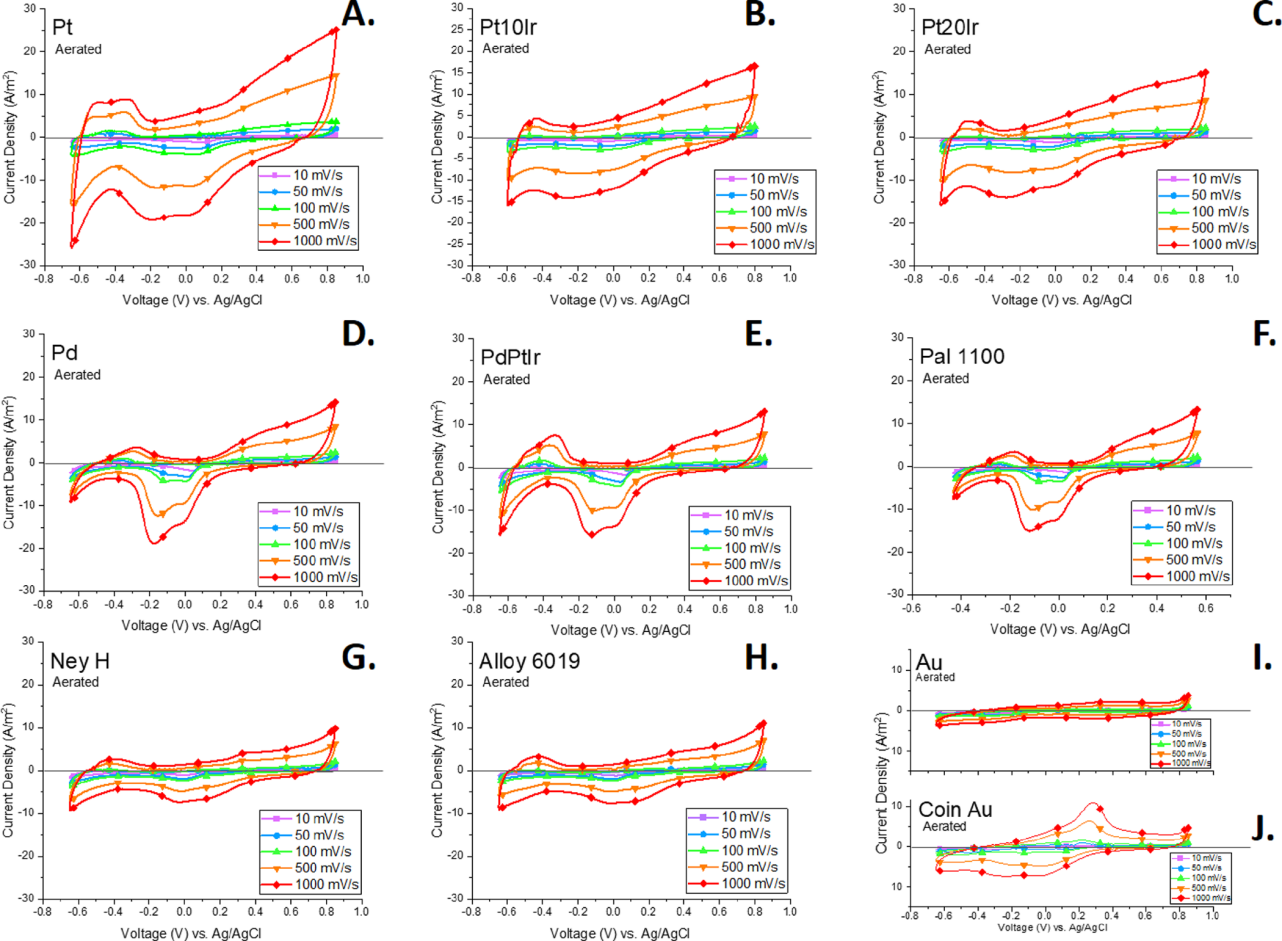
CV scans done under aerated conditions for all electrode materials at 10 (purple square), 50 (blue circle), 100 (green triangle), 500 (orange downward triangle), and 1000 (red diamond) mV/s scan rates. Pt (A), Pt10Ir (B), Pt20Ir (C), Pd (D), PdPtIr (E), Pal 1100 (F), Ney H (G), Alloy 6019 (H), Au (I), and Coin Au (J) are shown. Note the differences in Au and Coin Au *y*-axis ranges.

Figure 5 depicts CV scans done under aerated conditions at five different scan rates ranging from 10 to 1000 mV/s. As the scan rate increases, the peak current densities reached at each voltage increase proportionally for all materials. Oxide reduction, hydrogen adsorption, hydrogen desorption, and oxide formation peaks occur at similar voltages but become broader and less defined as the scan rate increases for all materials. Calculations for each peak were carried out for each scan rate of each material and are shown in Table 4.

**Table 4:** Summary of CSC Values for Each CV Scan Peak, the H Reversibility %, and CSC_H_ Value at Each Scan Rate Tested in the Aerated Condition

measurement	Pt	Pt10Ir	Pt20Ir	Au^a^	Coin Au^a^	Ney H	Alloy 6019	Pd	Pal 1100	PdPtIr
10 mV/s										
CSC_OxideRed_ (mC/cm^2^)	–4.23 ± 0.21	–4.13 ± 0.11	–4.02 ± 0.3	–1.03 ± 0.10	–3.18 ± 0.05	–2.25 ± 0.50	–3.58 ± 0.15	–3.56 ± 0.05	–4.28 ± 0.13	–3.43 ± 0.11
CSC_HA_ (mC/cm^2^)	–0.34 ± 0.03	–0.23 ± 0.01	–0.16 ± 0.01	N/A	N/A	–0.42 ± 0.02	–0.15 ± 0.01	–0.60 ± <0.01	–0.64 ± 0.02	–0.55 ± 0.02
CSC_HD_ (mC/cm^2^)	0.45 ± 0.04	0.21 ± 0.06	0.21 ± <0.01	0.20 ± 0.08	0.41 ± 0.04	0.32 ± 0.09	0.16 ± 0.02	0.67 ± 0.02	0.88 ± 0.09	0.71 ± 0.04
CSC_OxideForm_ (mC/cm^2^)	2.96 ± 0.03	3.10 ± 0.09	2.49 ± 0.40	N/A	N/A	1.23 ± 0.08	1.37 ± 0.07	1.73 ± 0.05	2.42 ± 0.06	2.17 ± 0.10
H reversibility %	75.0 ± 8.9	76.7 ± 8.8	79.9 ± 6.1	N/A	N/A	73.5 ± 17.9	83.2 ± 3.3	89.1 ± 3.1	73.4 ± 4.8	77.8 ± 6.2
CSC_H_ (mC/cm^2^)	0.79 ± 0.05	0.45 ± 0.06	0.37 ± 0.02	N/A	N/A	0.74 ± 0.09	0.31 ± 0.02	1.27 ± 0.02	1.52 ± 0.11	1.26 ± 0.03
50 mV/s										
CSC_OxideRed_ (mC/cm^2^)	–1.63 ± 0.11	–1.60 ± 0.09	–1.56 ± 0.16	–1.87 ± 0.15	–2.11 ± 0.09	–0.91 ± 0.06	–0.36 ± 0.02	–1.55 ± 0.02	–2.01 ± 0.10	–1.47 ± 0.09
CSC_HA_ (mC/cm^2^)	–0.31 ± 0.02	–0.17 ± <0.01	–0.13 ± 0.01	N/A	N/A	–0.43 ± 0.07	–0.17 ± 0.01	–0.69 ± 0.04	–0.72 ± 0.02	–0.78 ± 0.03
CSC_HD_ (mC/cm^2^)	0.28 ± 0.01	0.15 ± 0.01	0.12 ± 0.01	0.32 ± 0.04	0.64 ± 0.08	0.14 ± 0.05	0.08 ± <0.01	0.23 ± 0.04	0.34 ± 0.05	0.33 ± 0.04
CSC_OxideForm_ (mC/cm^2^)	0.81 ± 0.19	1.08 ± 0.06	0.81 ± 0.19	N/A	N/A	1.08 ± 0.05	1.10 ± 0.05	1.11 ± 0.02	1.66 ± 0.16	1.21 ± 0.07
H reversibility %	88.5 ± 7.2	85.7 ± 3.0	90.2 ± 8.5	N/A	N/A	31.3 ± 6.9	48.4 ± 3.9	32.4 ± 4.4	47.5 ± 5.9	42.5 ± 4.7
CSC_H_ (mC/cm^2^)	0.59 ± <0.01	0.32 ± 0.01	0.25 ± 0.02	N/A	N/A	0.56 ± 0.11	0.26 ± 0.01	0.92 ± 0.08	1.07 ± 0.07	1.11 ± 0.06
100 mV/s										
CSC_OxideRed_ (mC/cm^2^)	–1.04 ± 0.05	–0.99 ± 0.07	–0.93 ± 0.07	–1.08 ± 0.14	–1.64 ± 0.16	–0.54 ± 0.06	–0.13 ± 0.03	–1.11 ± 0.03	–1.44 ± 0.09	–0.98 ± 0.07
CSC_HA_ (mC/cm^2^)	–0.31 ± 0.04	–0.16 ± 0.01	–0.12 ± 0.01	N/A	N/A	–0.28 ± 0.05	–0.13 ± 0.01	–0.42 ± 0.02	–0.46 ± 0.02	–0.54 ± 0.01
CSC_HD_ (mC/cm^2^)	0.23 ± 0.02	0.13 ± <0.01	0.09 ± 0.01	0.28 ± 0.06	0.72 ± 0.14	0.09 ± 0.03	0.08 ± <0.01	0.13 ± <0.01	0.26 ± 0.01	0.28 ± 0.05
CSC_OxideForm_ (mC/cm^2^)	0.54 ± 0.08	0.74 ± 0.09	0.60 ± 0.09	N/A	N/A	0.67 ± 0.08	0.90 ± 0.04	0.87 ± 0.05	1.22 ± 0.02	0.81 ± 0.05
H reversibility %	74.2 ± 3.2	82.0 ± 3.3	79.9 ± 9.0	N/A	N/A	31.2 ± 3.9	58.3 ± 2.8	31.7 ± 2.0	55.7 ± 1.0	50.7 ± 8.6
CSC_H_ (mC/cm^2^)	0.54 ± 0.06	0.29 ± 0.01	0.21 ± 0.02	N/A	N/A	0.37 ± 0.07	0.21 ± 0.02	0.55 ± 0.02	0.71 ± 0.04	0.82 ± 0.05
500 mV/s										
CSC_OxideRed_ (mC/cm^2^)	–0.50 ± 0.07	–0.39 ± 0.01	–0.41 ± 0.02	–0.42 ± 0.07	–0.81 ± 0.08	–0.18 ± 0.05	–0.17 ± 0.01	–0.58 ± 0.02	–0.76 ± 0.07	–0.50 ± 0.03
CSC_HA_ (mC/cm^2^)	–0.20 ± 0.03	–0.09 ± 0.01	–0.05 ± <0.01	N/A	N/A	–0.10 ± 0.03	–0.07 ± 0.01	–0.11 ± 0.01	–0.13 ± <0.01	–0.23 ± <0.01
CSC_HD_ (mC/cm^2^)	0.20 ± 0.03	0.07 ± <0.01	0.05 ± <0.01	0.21 ± 0.04	0.56 ± 0.09	0.04 ± 0.02	0.03 ± 0.01	0.06 ± 0.01	0.11 ± 0.01	0.15 ± 0.02
CSC_OxideForm_ (mC/cm^2^)	0.50 ± 0.05	0.38 ± 0.05	0.27 ± 0.05	N/A	N/A	0.24 ± 0.03	0.34 ± 0.05	0.42 ± 0.01	0.73 ± 0.10	0.46 ± 0.06
H reversibility %	96.1 ± 2.5	85.6 ± 5.5	95.1 ± 3.6	N/A	N/A	43.9 ± 5.8	44.2 ± 8.6	52.2 ± 10.2	80.3 ± 3.8	66.2 ± 5.3
CSC_H_ (mC/cm^2^)	0.40 ± 0.06	0.16 ± 0.01	0.11 ± 0.01	N/A	N/A	0.14 ± 0.04	0.11 ± 0.02	0.17 ± <0.01	0.24 ± 0.01	0.38 ± 0.02
1000 mV/s										
CSC_OxideRed_ (mC/cm^2^)	–0.62 ± 0.05	–0.26 ± 0.02	–0.25 ± <0.01	–0.31 ± 0.07	–0.65 ± 0.09	–0.14 ± 0.04	–0.14 ± 0.01	–0.45 ± <0.01	–0.60 ± 0.02	–0.37 ± 0.02
CSC_HA_ (mC/cm^2^)	–0.13 ± 0.01	–0.05 ± 0.01	–0.03 ± <0.01	N/A	N/A	–0.06 ± 0.02	–0.05 ± 0.01	–0.05 ± 0.01	–0.06 ± <0.01	–0.15 ± <0.01
CSC_HD_ (mC/cm^2^)	0.15 ± 0.02	0.05 ± <0.01	0.03 ± <0.01	0.19 ± 0.03	0.49 ± 0.09	0.03 ± 0.02	0.04 ± 0.01	0.04 ± 0.01	0.06 ± <0.01	0.11 ± <0.01
CSC_OxideForm_ (mC/cm^2^)	0.34 ± 0.04	0.32 ± 0.06	0.30 ± 0.03	N/A	N/A	0.25 ± 0.03	0.29 ± 0.03	0.41 ± 0.03	0.61 ± 0.07	0.40 ± 0.01
H reversibility %	91.2 ± 5.3	91.8 ± 3.2	89.2 ± 8.8	N/A	N/A	41.4 ± 16.9	70.7 ± 0.3	85.1 ± 4.1	89.4 ± 2.9	75.1 ± 0.9
CSC_H_ (mC/cm^2^)	0.28 ± 0.03	0.10 ± 0.01	0.06 ± 0.01	N/A	N/A	0.09 ± 0.04	0.09 ± 0.02	0.09 ± 0.01	0.12 ± <0.01	0.25 ± <0.01

a Individual peak values were not calculated for Au and Coin Au. The Au and Coin Au CSC_C_ and CSC_A_ values are listed here under CSC_OxideRed_ and CSC_HD_, respectively.

**Figure 6:**
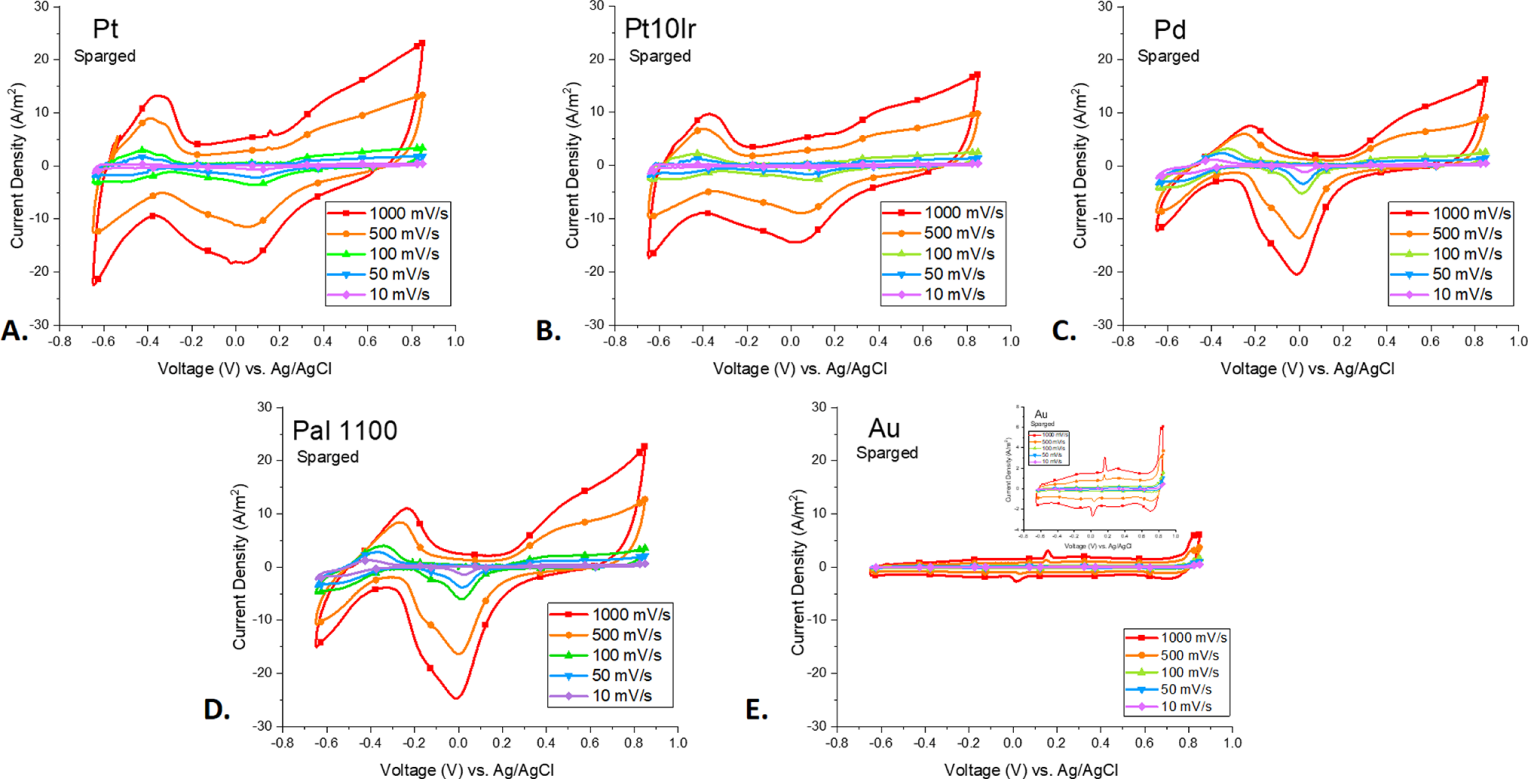
CV scans done under sparged conditions for Pt (A), Pt10Ir (B), Pd (C), Pal 1100 (D), and Au (E) at 10 (purple diamond), 50 (blue downward triangle), 100 (green triangle), 500 (orange circle), and 1000 (red square) mV/s scan rates.

Similarly, Figure 6 depicts CV scans done under sparged conditions at five different scan rates, from 10 to 1000 mV/s. Again, an increase in current densities and peak broadening is seen as the scan rate is increased. Table 5 shows calculations for each peak at each scan rate for all materials studied in the sparged condition.

**Table 5:** Summary of CSC Values for Each CV Scan Peak, the Reversibility % of H Adsorption/Desorption, and Total H CSC Value at Each Scan Rate Tested in the Sparged Condition

	measurement	Pt	Pt10Ir	Au^a^	Pd	Pal 1100
10 mV/s	CSC_OxideRed_ (mC/cm^2^)	–1.34 ± 0.17	–1.16 ± 0.31	–0.18 ± 0.07	–1.11 ± 0.26	–1.54 ± 0.42
	CSC_HA_ (mC/cm^2^)	–0.40 ± 0.05	–0.27 ± 0.04	N/A	–1.81 ± 0.13	–2.27 ± 0.11
	CSC_HD_ (mC/cm^2^)	0.48 ± 0.08	0.39 ± 0.03	0.25 ± 0.03	1.78 ± 0.09	2.08 ± 0.14
	CSC_OxideForm_ (mC/cm^2^)	1.26 ± 0.16	0.57 ± 0.12	N/A	0.29 ± 0.08	0.68 ± 0.1
	H reversibility %	85.4 ± 18.6	68.8 ± 5.7	N/A	96.9 ± 2.7	91.7 ± 4.2
	CSC_H_ (mC/cm^2^)	0.88 ± 0.03	0.67 ± 0.07	N/A	3.59 ± 0.21	4.35 ± 0.23
50 mV/s	CSC_OxideRed_ (mC/cm^2^)	–0.78 ± 0.08	–0.66 ± 0.11	–0.36 ± 0.02	–0.79 ± 0.16	–1.12 ± 0.24
	CSC_HA_ (mC/cm^2^)	–0.36 ± 0.08	–0.30 ± 0.02	N/A	–0.83 ± 0.06	–0.83 ± 0.04
	CSC_HD_ (mC/cm^2^)	0.44 ± 0.11	0.26 ± 0.05	0.34 ± 0.02	0.60 ± 0.03	0.68 ± 0.05
	CSC_OxideForm_ (mC/cm^2^)	0.91 ± 0.10	0.49 ± 0.13	N/A	0.62 ± 0.07	1.05 ± 0.19
	H reversibility %	79.9 ± 12.8	85.6 ± 9.1	N/A	72.4 ± 1.8	82.8 ± 4.7
	CSC_H_ (mC/cm^2^)	0.80 ± 0.18	0.56 ± 0.06	N/A	1.44 ± 0.09	1.51 ± 0.08
100 mV/s	CSC_OxideRed_ (mC/cm^2^)	–0.67 ± 0.08	–0.53 ± 0.06	–0.33 ± 0.02	–0.73 ± 0.15	–0.98 ± 0.2
	CSC_HA_ (mC/cm^2^)	–0.40 ± 0.06	–0.27 ± 0.02	N/A	–0.58 ± 0.03	–0.58 ± 0.01
	CSC_HD_ (mC/cm^2^)	0.45 ± 0.06	0.35 ± 0.04	0.28 ± 0.04	0.37 ± 0.02	0.50 ± 0.03
	CSC_OxideForm_ (mC/cm^2^)	1.08 ± 0.09	0.82 ± 0.17	N/A	0.57 ± 0.06	0.86 ± 0.17
	H reversibility %	87.7 ± 7.6	76.4 ± 5.4	N/A	65.0 ± 5.3	86.7 ± 5.7
	CSC_H_ (mC/cm^2^)	0.85 ± 0.12	0.62 ± 0.06	N/A	0.95 ± 0.02	1.08 ± 0.03
500 mV/s	CSC_OxideRed_ (mC/cm^2^)	–0.43 ± 0.08	–0.31 ± 0.04	–0.27 ± 0.03	–0.53 ± 0.10	–0.70 ± 0.11
	CSC_HA_ (mC/cm^2^)	–0.25 ± 0.02	–0.14 ± 0.01	N/A	–0.29 ± 0.03	–0.32 ± 0.01
	CSC_HD_ (mC/cm^2^)	0.27 ± 0.03	0.18 ± 0.01	0.23 ± 0.03	0.19 ± 0.02	0.26 ± 0.02
	CSC_OxideForm_ (mC/cm^2^)	0.58 ± 0.07	0.51 ± 0.07	N/A	0.53 ± 0.11	0.74 ± 0.12
	H reversibility %	84.4 ± 2.2	80.1 ± 5.4	N/A	67.4 ± 10.5	80.1 ± 5.8
	CSC_H_ (mC/cm^2^)	0.52 ± 0.03	0.32 ± 0.01	N/A	0.48 ± 0.03	0.58 ± 0.03
1000 mV/s	CSC_OxideRed_ (mC/cm^2^)	–0.11 ± 0.23	–0.21 ± 0.01	–0.24 ± 0.02	–0.45 ± 0.09	–0.55 ± 0.1
	CSC_HA_ (mC/cm^2^)	–0.17 ± 0.01	–0.09 ± 0.01	N/A	–0.16 ± 0.02	–0.17 ± 0.01
	CSC_HD_ (mC/cm^2^)	0.18 ± 0.04	0.11 ± <0.01	0.22 ± 0.01	0.13 ± 0.02	0.16 ± <0.01
	CSC_OxideForm_ (mC/cm^2^)	0.46 ± 0.03	0.39 ± 0.07	N/A	0.44 ± 0.09	0.60 ± 0.08
	H reversibility %	83.7 ± 8.3	84.0 ± 4.3	N/A	72.3 ± 11.7	92.1 ± 4.2
	CSC_H_ (mC/cm^2^)	0.35 ± 0.04	0.20 ± 0.01	N/A	0.28 ± 0.01	0.33 ± 0.02

a Individual peak values were not calculated for Au. The Au CSC_C_ and CSC_A_ values are listed here under CSC_OxideRed_ and CSC_HD_, respectively.

**Figure 7:**
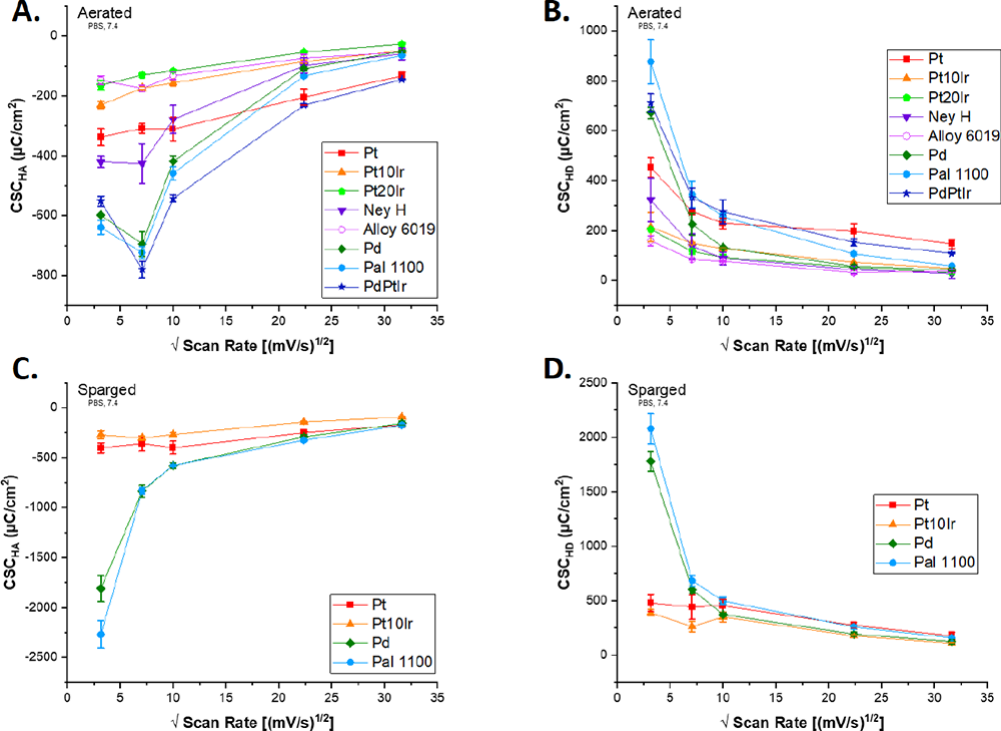
(A) Aerated CSC_HA_ vs square root of scan rate. (B) Aerated CSC_HD_ value plotted vs square root of scan rate. **(**C) Sparged CSC_HA_ vs square root of scan rate. (D) Sparged CSC_HD_ value plotted vs square root of scan rate. CSC_HA_ and CSC_HD_ values not calculated for Au and Coin Au.

Figure 7 compares the CSC_HA_ and CSC_HD_ values for each electrode material versus scan rate for both aerated and sparged environments. Overall, as scan rate increases, the magnitudes of CSC_HA_ and CSC_HD_ to both hydrogen absorption and desorption both decrease.

## Discussion

### Effect of the Electrode Material

The platinum-based electrodes all demonstrated the classic sequence of oxide reduction, hydrogen adsorption, H_2_ formation, hydrogen desorption, oxide formation, and O_2_ formation peaks in the potential window tested (Figures 2 and 3). The size and definition of these peaks decreased with increasing iridium weight percent. Just like Pt, Ir is known to demonstrate two hydrogen adsorption peaks in the cathodic scan, with the higher potential peak associated with strongly adsorbed hydrogen and the lower potential peak associated with weaker hydrogen adsorption. It is known that the Ir surface becomes covered with more of the weakly adsorbed hydrogen than Pt, which contains a higher percentage of the strongly adsorbed hydrogen.^25^ This may explain the decrease in peak size seen with increasing Ir weight percent. This signifies that there is an engineering tradeoff in stimulation electrode design: the use of Ir as a solid solution strengthener in Pt is well known, but its use decreases CSC of the alloy.

The Pd family of electrodes shows a single, broad hydrogen adsorption peak at −0.5 V that then immediately forms the start of the H_2_ formation peak, making the two peaks hard to distinguish in the aerated scans. This peak elongation and lack of perfect symmetry in the anodic scan may be the result of hydrogen penetration into the Pd lattice, as H is known to have significant solid solubility in Pd. Atomic H that migrates into the Pd lattice would not necessarily be available for the desorption reaction in the Pd analog to eq 3. The rate limiting step in the hydrogen absorption process is the hydrogen penetration into the Pd surface; this kinetic effect is discussed further below.^26^ Neither the addition of Re in Pal 1100 nor the addition of Pt and Ir in PdPtIr had a significant effect on the shape of the CV curve. Unlike the addition of Ir to Pt, the addition of Re to Pd acted to increase CSC_H_ but decreased the reversibility of hydrogen sorption.

The Au family of electrodes was heavily influenced by each of their additional constituents. The CV scan of pure Au is highly asymmetric with a possible oxide formation peak in the anodic scan and a known oxide reduction peak in the cathodic scan (Figure S2).^27,28^ The Au scan had several distinct peaks in the cathodic scan dissimilar to those seen in the Pt and Pd scans, and no supporting literature could be found to suggest hydrogen adsorption. Alloy 6019, which contains Au (majority), Pd, Pt, and Ir, shows what appears to be an oxide reduction peak and then a hydrogen adsorption peak both smaller than those seen in the Pt and Pd families. However, the anodic scan is similar to that of Au. Ney H, similar to 6019 but with a higher Pd and lower Pt content, is very similar to the Pd scan with smaller peaks in both directions. Coin Au appears very similar to Au with the addition of nearly symmetric anodic and cathodic peaks at 0.2 V. A scanning window of 0.8 to −0.65 V was chosen here for material comparison; however, it is important to note the known oxidation and reduction peaks that occur for gold materials outside of this window.^28^ It is possible that the gold materials may have fared better in the material rankings had a different scanning region been chosen for comparison, an interesting topic for future investigation.

### Aerated vs Sparged Condition

Comparing material performance in both an aerated (oxygen rich) and a sparged (hypoxic or limited oxygen) environment is important for understanding how materials will behave in biological milieu, where tissues have variable oxygen content. Oxygen pressure in air (160 mmHg) is greater than that in specific intestinal tissues (57.6 ± 2.3 mmHg) or in the brain (33.8 ± 2.6 mmHg). Oxygen partial pressures can fluctuate in the body depending on tissue requirements, function, and stasis.^29^ In this report, we have chosen to study electrode performance at the extreme ends of oxygen concentrations in tissues to study the effects oxygen partial pressure in the microenvironment might have on electrochemical reactions happening at the surface of each type of electrode.

When oxygen was present in the electrode cell, oxygen reduction was observed at all voltages less than 0.2 V.^12,30^ The oxygen reduction reaction current superimposes the hydrogen adsorption and desorption reactions.^31^ For this reason, a decrease is observed in the current baseline during the cathodic sweep.^12^ The aerated condition resulted in lower overall CSC_H_ values for each material than the sparged condition. However, both aerated and sparged conditions yielded the same material ranking for CSC_H_: Pal 1100, Pd, Pt, Pt10Ir, and then Au.

H reversibility % values were similar for both aerated and sparged conditions, suggesting that it is not significantly affected by the presence of oxygen, an encouraging finding for applications in bodily tissues that maintain fluctuating oxygen partial pressures.

CSC_TOTAL_ values were larger under aerated conditions than sparged conditions, driven by larger oxide formation and reduction peaks seen in the presence of oxygen. Like H reversibility %, total reversibility between aerated and sparged conditions was not wildly different; however, in general, the sparged value tended to be higher. This may have been an effect of the baseline changes caused by the presence of oxygen.

It is important to note that any differences seen between aerated and sparged scans in the region greater than 0.2 V may be due to the averaging of several trials between each material as well as differences in pH caused by different oxygen partial pressures or the gas pressure variation between aerated and sparged conditions.^32,33^ These slight variations are insignificant and do not affect the evaluation of the materials or any resulting conclusions.

### Effect of Scan Rate

The effect of increasing scan rate on material performance is important to determine due to the nature of the short current pulses used in neuronal stimulation that do not necessarily allow all faradaic processes to occur.^34^ Increasing scan rates caused an increase in pseudo-capacitive currents, resulting in the increased peak current values seen in Figures 5 and 6.^35^ Conversely, charge storage capacity values decreased as scan rate increased. This, combined with the visible peak broadening at higher scan rates, was largely due to a decrease in time available for electrolytes to adsorb onto the available electrode surface areas and then desorb completely.^36^

**Figure 8:**
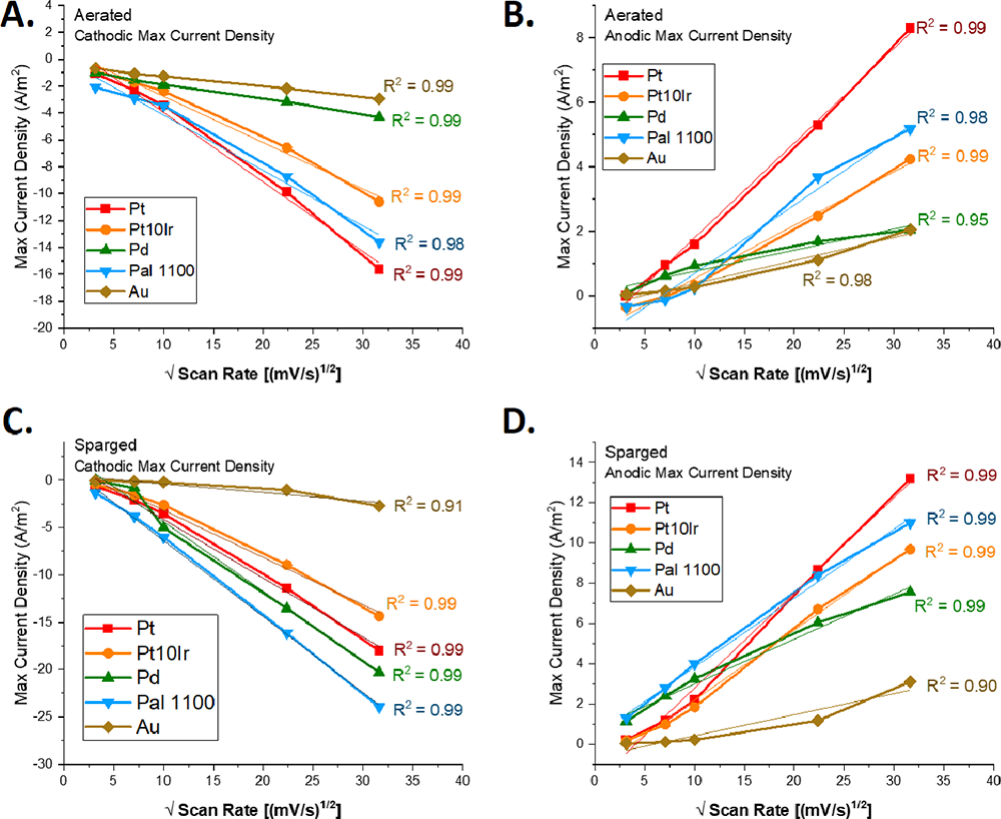
Linear regression for selected materials of peak current density as a function of root scan rate. Aerated, cathodic (A), aerated, anodic (B), sparged, cathodic (C), and sparged, anodic (D) peak current densities for Pt, Pt10Ir, Pd, Pal 1100, and Au all demonstrated a linear relationship with root scan rate.

All materials tested exhibited peak current densities with a nominally linear dependence on the square root of scan rate, as shown in Figure 8. This linear dependence suggests that a diffusion-controlled process dominates the material surface reactions in the studied systems. A similar correlation was noted previously by others, for instance, Alkhalaf *et al*.^36^

Some material CSC values were less affected by higher scan rates than others (Figure 7). Pd-based alloys showed large decreases in absolute CSC_HA_ and CSC_HD_ values when scan rates greater than 10 mV/s were employed. This points toward slower reaction kinetics at the Pd surface, possibly due to the aforementioned hydrogen absorption. Hydrogen diffusion into Pd occurs at a rate of 10^–11^ m^2^/s, after first adsorbing onto the surface and then transferring through to the subsurface (the rate-determining step).^37^

One possible interpretation is that faster scan rates limit the completion of the rate-determining transfer step needed for H absorption into Pd and its alloys. In that case, one would expect higher scan rates to decrease absolute values of CSC_HA_ and CSC_HD_. However, a concurrent increase in H reversibility would also be expected as limited H transfer into solid Pd would eliminate the potential parasitic effect of H absorption on reversibility. In fact, the H reversibility, CSC_HA_, and CSC_HD_ all decrease with increasing scan rate, suggesting some other deficiency of Pd-based materials at higher scan rates. This effect requires further study.

PBS was chosen in this work as the media to mimic the buffering capacity of human tissues. It should be considered here that the cathodic scan is more heavily influenced by a buffering environment than the anodic scan. The cathodic scan involves oxide reduction and hydrogen adsorption, which are both limited by proton availability and diffusion, directly affected by diffusion of all components of the buffering system. The anodic scan, involving hydrogen desorption and oxide formation, is thought to be solely limited by reaction kinetics.^14^Figure S3 plots the difference between CSC_C_ and CSC_A_ values calculated for scan rates of 10 mV/s vs 1000 mV/s. In all materials tested, the change in the CSC_C_ value was greater than the change in CSC_A_ when the two scan rates are compared.

Slower scan rates allow more gradual sequestering and release of hydrogen on the metal surface than faster scan rates. The latter may result in a “burst release” of H upon desorption. Therefore, it is expected that higher scan rates may produce some localized pH fluctuation and buffering kinetics will dictate the extent to which local pH may vary as surface reactions occur. This could be a contributor to the largely diminished CSC_HA_ and CSC_HD_ values at higher scan rates for all tested materials.^14^ This effect is well known on very high surface area electrodes and is something to consider in the design of both materials and *in vitro* experiments. In future work, the use of a simulated body fluid (SBF) that more closely mimics biological buffering through a combination of bicarbonate, phosphate, and organic acids may offer a more faithful reproduction of the physiological environment.

### Comparison of Material for Use in Tissue Stimulation

Suitability of materials to bioelectrode application was ranked on a relative basis in Table 6. Comparison was made based on the following categories under aerated conditions at 10 mV/s: CSC_H_, H reversibility %, CSC_TOTAL_, and total reversibility %. The material with the highest value in a category is considered to be the “best” and is assigned a 1, the second highest value assigned a 2, and so on. The final column in Table 6 is a sum of each material’s ranking for the preceding columns. A lower sum indicates that the material had a relatively good suitability as an electrode material based on criteria considered in this work. Here, we consider that a material with increased hydrogen charge storage as well as increased reversibility of hydrogen charge storage will perform better as an implantable electrode due to its ability to inject increased electrical charge into the tissue without irreversible electrochemical reactions occurring. Using this scheme, Pt and Pal 1100 tied as the best-performing materials (lowest sum) in the aerated condition. They were followed closely by Pt10Ir and PdPtIr, which tied as the next-lowest sum.

**Table 6:** Material Rankings for CSC_H_, H Reversibility %, CSC_TOTAL_, and Total Reversibility % at 10 mV/s Are Tabulated for the Aerated Condition^a^

	rank CSC_H_	rank H rev %	rank CSC_TOTAL_	rank total rev %	sum
Pt	4	6	2	2	14
Pal 1100	1	8	1	4	14
Pt10Ir	6	5	3	1	15
PdPtIr	3	4	5	3	15
Pd	2	1	6	7	16
Pt20Ir	7	3	4	5	19
Alloy 6019	8	2	7	8	25
Ney H	5	7	8	6	26
Au	9	9	10	9	37
Coin Au	9	9	9	10	37

a “1” indicates the highest (best) value in that category. “10” indicates the lowest (worst) value. Materials are organized by the lowest total ranking sum.

Table 7 mimics Table 6 for the sparged condition. Pal 1100 performed the best for these specified categories in the sparged condition followed by Pd, Pt, Pt10Ir, and Au.

**Table 7:** Material Rankings for CSC_H_, H Reversibility %, CSC_TOTAL_, and Total Reversibility % at 10 mV/s Are Tabulated for the Sparged Condition, Using the Same Ranking Method as Table 6

	rank CSC_H_	rank H rev %	rank CSC_TOTAL_	rank total rev %	sum
Pal 1100	1	2	1	3	7
Pd	2	1	2	4	9
Pt	3	3	3	1	10
Pt10Ir	4	4	4	5	17
Au	5	5	5	2	17

**Figure 9:**
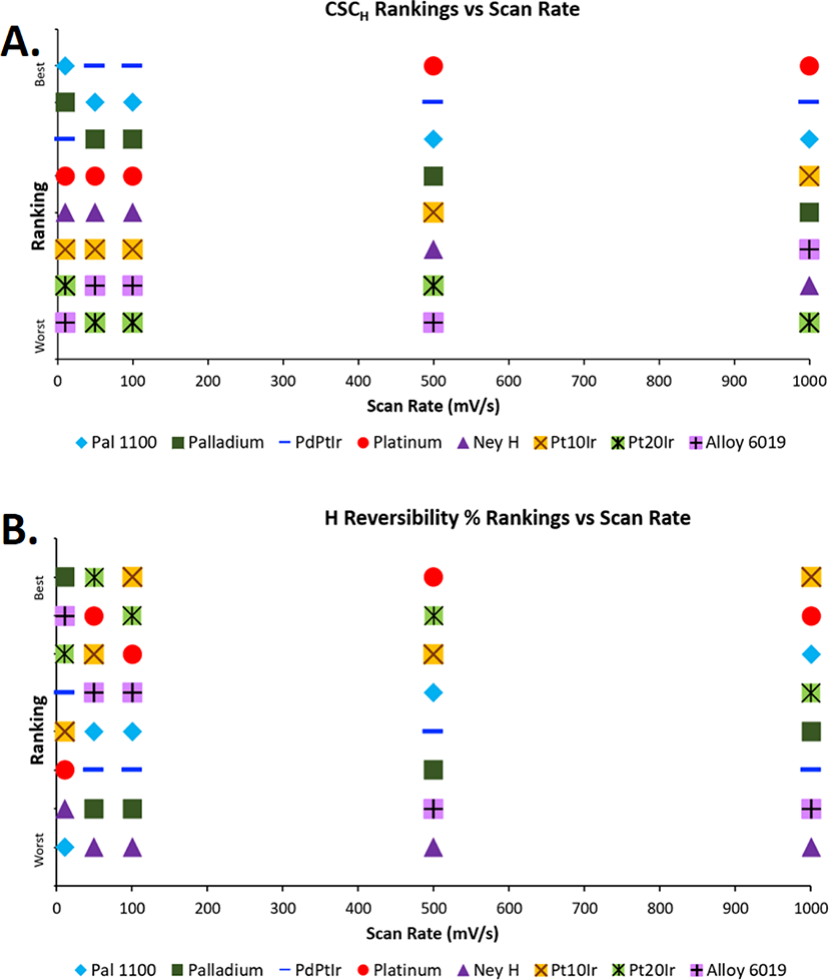
(A) CSC_H_ rankings vs scan rate. (B) H reversibility % rankings vs scan rate.

Figure 9 visually represents the CSC_H_ and H reversibility % rankings for materials in the aerated condition at each scan rate. Pd-based electrodes earned the top spots at the lower scan rates for CSC_H_ values, with Pt overtaking Pd and its alloys at higher scan rates of 500 and 1000 mV/s. Figure 9B makes it clear that platinum-based electrodes perform better at all scan rates in H reversibility percentages. However, Pal 1100 ranks third at the highest scan rate.

**Figure 10:**
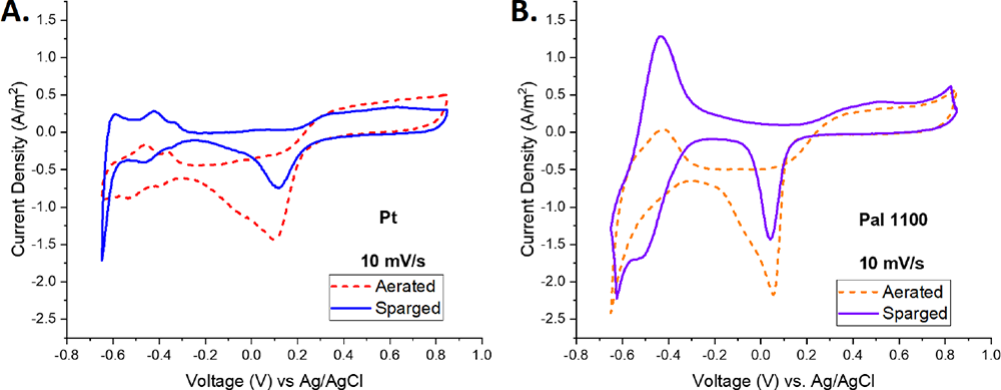
Comparison of the Pt (A) and Pal 1100 (B) electrodes in the aerated vs sparged conditions at a 10 mV/s scan rate.

Figure 10 shows a close-up comparison of the top-performing platinum and Pal 1100 electrodes at 10 mV/s in the aerated and sparged conditions. At this scale, the differences in peak shape and heights between these two materials become more apparent. H_ads_, H_des_, and oxide reduction peak areas are significantly larger in the Pal 1100 CV scans relative to the platinum scans. Sparged Pal 1100 has a larger CSC_HA_ before the evolution of H_2_; however, it lacks the symmetry seen in the Pt electrode’s overall behavior, resulting in lower reversibility percentages.

A number of tissue stimulation electrodes available on the market are made with Pt and Pt10Ir. However, there is very little information on the Pd electrode material use in tissue stimulation applications.

These results point toward Pt, Pd, and their alloys being plausible materials for TCEs, based on their higher CSC values due to generally reversible electrochemical reactions. However, the utility of Pd as an electrode material is not reflected in implantable electrodes available on the market. Because Pd alloys may be fabricated with mechanical properties that exceed Pt10Ir, this asymmetry is not likely due to structural material characteristics. Other factors may be cost volatility, availability, relative regulatory hurdles, and possibly an early and well-entrenched bias toward Pt materials. Palladium’s ability to absorb hydrogen and lower reversibility may also present barriers to application. Several reports have shown that engineering of the Pd material to vary grain size, lattice parameter, and alloying constituents may accelerate hydrogen sorption rates.^37^ Results shown here agree that these are worthwhile endeavors, and Pd-based materials should be considered as alternative implantable electrode materials.

## Conclusions

Ten different noble metals and alloys were compared as tissue contacting electrode materials using CV. Both aerated and sparged conditions were tested for a subset of materials. CSC values were calculated for each peak in each CV scan to better understand the electrochemical reactions occurring at each material’s surface. Total CSC values and reversibility percentage values were calculated for each material to help predict and compare material efficacy in vivo. Pt and Pal 1100 (Pd-Re) electrodes consistently ranked highest, in terms of total charge storage due to hydrogen adsorption and desorption and total electrochemical reaction reversibility. Tests under aerated and sparged (hypoxic) conditions did not reveal large differences in relative material suitability for bioelectrodes. At lower scan rates of 10, 50, and 100 mV/s, the palladium-based electrodes achieved the highest CSCs based on hydrogen sorption; platinum-based electrodes exhibited the highest hydrogen sorption reversibility at higher scan rates of 500 and 1000 mV/s.

## Supporting Information Available

Electrode compositions, anodic vs cathodic changes in CSC values with increasing scan rate, and example surface roughness measurement (PDF)
