# Supplementary material for: Cyclic Voltammetry Study of Noble Metals and Their Alloys for Use in Implantable Electrodes
Source: ACS Omega. 2022 Sep 13;7(38):34200–12. doi: 10.1021/acsomega.2c03563 (PMC9520554; doi:10.1021/acsomega.2c03563)
Supplement: Supplementary file 1 — ao2c03563_si_001.pdf [file ao2c03563_si_001.pdf]

Supplemental Information

A Cyclic Voltammetry Study of Noble Metals and their Alloys for Use in Implantable Electrodes

Megan K. Puglia*, and Patrick K. Bowen

Research & Development, Deringer-Ney, Inc., 353 Woodland Ave, Bloomfield, CT, 06002

*Corresponding author: mpuglia@deringerney.com
KEYWORDS: implantable electrode, platinum, gold, palladium, charge storage capacity, reversibility.

Table S1. Electrode compositions as analyzed by XRF, solutions-based inductively coupled plasma optical emission spectroscopy (ICP-OES) and/or laser ablation ICP-OES.

Constituent	Au	Coin Au	6019	Ney H	Pt	Pt10Ir	Pt20Ir	Pd	Pal 1100	PdPtIr
Ag	ND	24	12	268	ND	142	ND	475	ND	321
Al	-	-	-	8	-	-	-	ND	84	192
As	ND	6	ND	ND	ND	ND	ND	ND	ND	ND
Au	>99.99 wt.%	99.01 wt.%	60.43 wt.%	57.74 wt.%	2	ND	ND	ND	ND	10
B	ND	ND	21	-	ND	ND	ND	247	600	15
Ba	-	-	-	-	-	-	-	ND	ND	ND
Be	ND	ND	ND	ND	-	ND	ND	ND	-	ND
Bi	ND	ND	ND	ND	ND	ND	ND	ND	1	ND
C	-	-	-	-	-	-	-	ND	-	ND
Ca	-	-	-	-	-	-	-	ND	ND	13
Cd	ND	ND	1	3	ND	ND	ND	ND	ND	ND
Co	ND	ND	3	2	ND	ND	4	ND	ND	ND
Cr	ND	ND	3	41	ND	44	41	ND	ND	ND
Cs	-	-	-	-	-	-	-	ND	-	ND
Cu	26	9.93 wt.%	ND	225	ND	10	ND	46	68	94
Fe	ND	12	3	7	15	27	11	15	ND	100
Ga	ND	ND	2	-	ND	ND	84	ND	ND	2
Ge	ND	ND	5	-	-	11	ND	ND	ND	ND
Hf	-	-	-	-	-	-	-	ND	-	ND
Hg	ND	ND	ND	3	ND	ND	ND	ND	-	ND
In	-	-	-	-	-	-	-	ND	ND	ND
Ir	ND	6	0.99 wt.%	0.95 wt.%	ND	10.08 wt.%	20.04 wt.%	ND	ND	0.97 wt.%
K	-	-	-	-	-	-	-	2	-	ND
Li	-	-	-	-	-	-	-	ND	-	ND
Mg	ND	ND	1	-	ND	ND	ND	ND	ND	ND
Mn	ND	ND	ND	-	ND	ND	ND	ND	ND	ND
Mo	ND	ND	ND	-	ND	ND	ND	ND	ND	ND
Na	-	-	-	-	-	-	-	-	-	ND
Nb	-	-	-	-	-	-	-	ND	-	ND
Ni	ND	247	ND	6	ND	27	ND	267	ND	ND
Os	ND	ND	ND	-	ND	ND	ND	35	ND	ND
P	-	-	-	-	-	-	-	ND	ND	ND
Pb	ND	ND	ND	37	ND	ND	ND	ND	ND	ND
Pd	6	131	19.92 wt.%	31.13 wt.%	ND	85	ND	99.89 wt.%	90.05 wt.%	78.99 wt.%
Pt	ND	16	18.66 wt.%	10.11 wt.%	>99.99 wt.%	89.88 wt.%	79.95 wt.%	ND	ND	20.04 wt.%
Re	ND	ND	ND	-	ND	ND	ND	ND	9.85 wt.%	ND
Rh	ND	ND	3	-	33	ND	ND	ND	ND	ND
Ru	ND	ND	ND	-	2	ND	ND	ND	ND	ND
S	-	-	-	-	-	-	-	ND	-	ND
Sb	ND	ND	ND	-	ND	ND	ND	ND	ND	ND
Se	-	-	-	-	-	-	-	ND	-	ND
Si	ND	172	ND	ND	ND	ND	ND	ND	ND	ND
Sn	ND	1	ND	34	ND	ND	ND	ND	7	ND
Sr	-	-	-	-	-	-	-	ND	-	ND
Ta	-	-	-	-	-	-	-	ND	-	ND
Te	-	-	-	-	-	-	-	ND	ND	ND
Ti	-	-	-	-	-	-	-	3	4	13
Tl	-	-	-	-	-	-	-	ND	-	ND
V	-	-	-	-	-	-	-	ND	-	ND
W	ND	ND	ND	31	-	ND	ND	25	ND	62
Zn	ND	ND	3	37	ND	ND	ND	3	ND	ND
Zr	ND	16	9	12	ND	5	2	ND	295	2

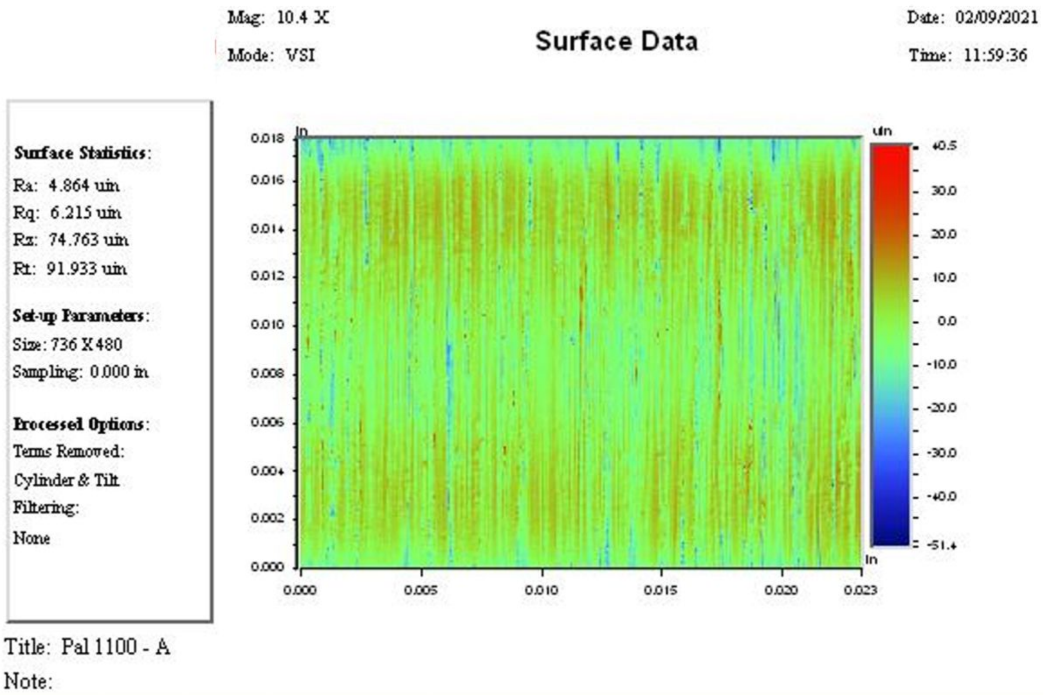

Figure S1. Interferometer surface roughness measurement image for Pal 1100 electrode A.

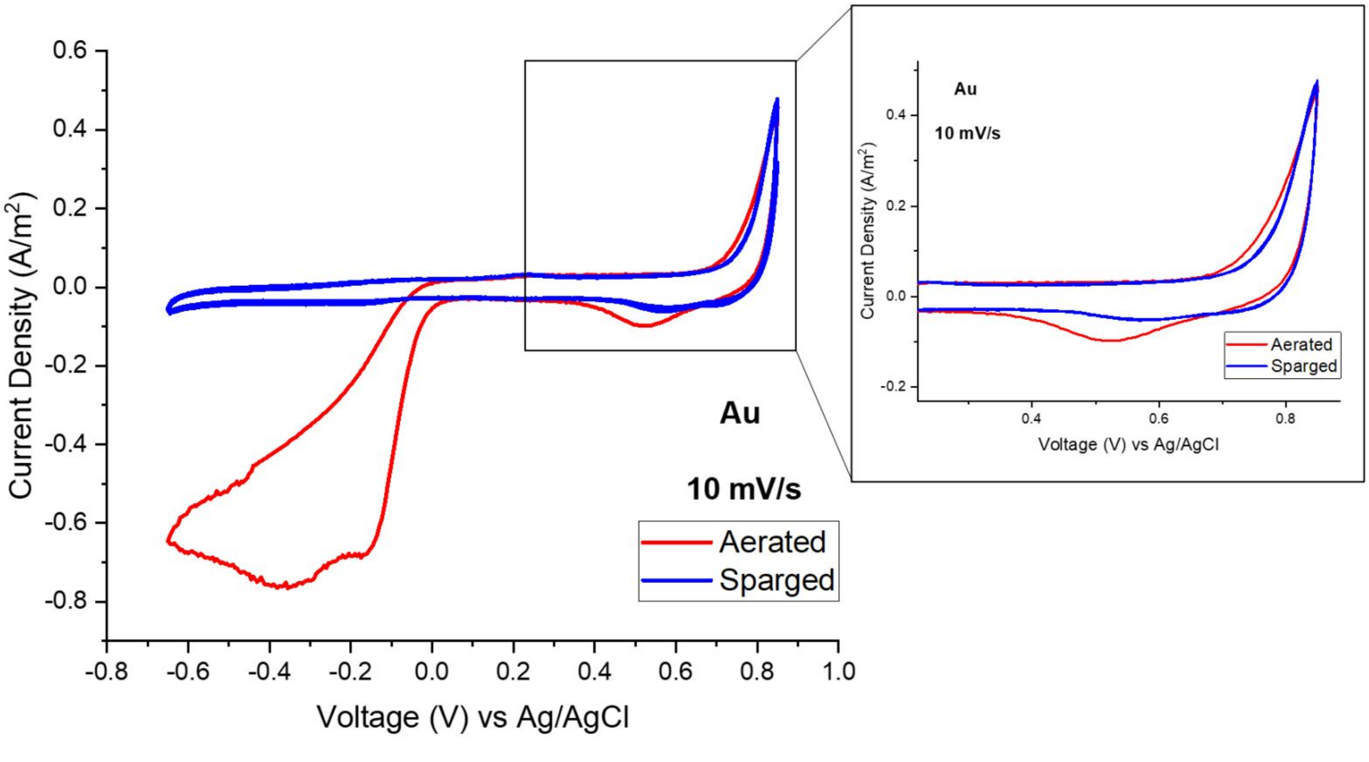

Figure S2. Close-up plot of pure Au electrode in both the aerated (red) and sparged (blue) conditions in 100 mM PBS. Inset shows a further zoomed-in view of the oxidation peak that begins to occur in both aerated and sparged anodic scans at about 0.75 V, as well as the reduction peak that occurs near 0.5 V in the cathodic scan.

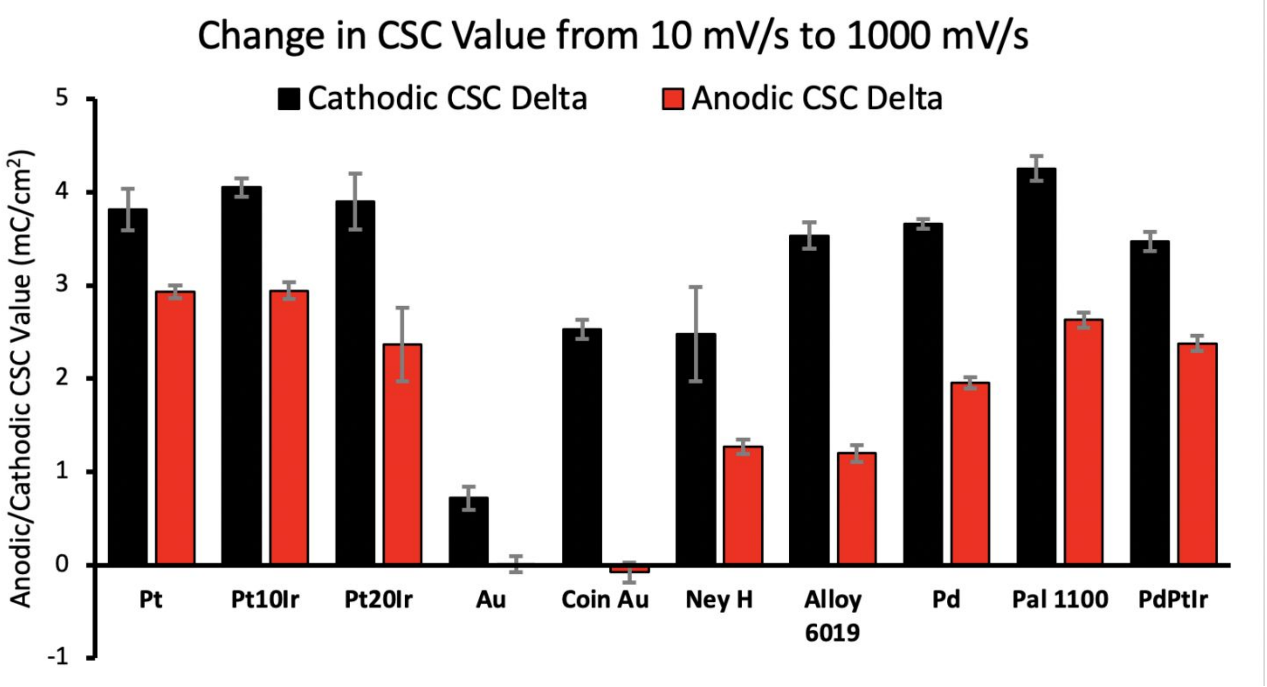

Figure S3. The difference in CSC_C and CSC_A values between 10 mV/s scans and 1000 mV/s scans for all materials in the aerated condition.
